# Supplementary material for: Excitation energy mediated cross-relaxation for tunable upconversion luminescence from a single lanthanide ion
Source: Nat Commun. 2022 Aug 12;13:4741. doi: 10.1038/s41467-022-32498-4 (PMC9374733; doi:10.1038/s41467-022-32498-4)
Supplement: Supplementary file 1 — Supplementary Information [file 41467_2022_32498_MOESM1_ESM.pdf]

Supplementary Materials for

Excitation energy mediated cross-relaxation for tunable upconversion luminescence from a single lanthanide ion

Xiao Fu,[1,7] Shuai Fu,[2,7] Qi Lu,[1,7] Jing Zhang,[2,7] Pingping Wan,[3] Jinliang Liu,[2] Yong Zhang,[4,] Chia-Hung Chen,[5] Wei Li,[1] Huadong Wang,[6] and Qingsong Mei,[1,6,*]*

[1] Department of Medical Biochemistry and Molecular Biology, School of Medicine, Jinan University, Guangzhou, Guangdong 510632, China

[2] School of Environmental and Chemical Engineering, Shanghai University, Shanghai 200444, China

[3] School of Food and Biological Engineering, Hefei University of Technology, Hefei, Anhui 230009, China

[4] Department of Biomedical Engineering, College of Design and Engineering, National University of Singapore, Singapore 117583, Singapore

[5] Department of Biomedical Engineering, City University of Hong Kong, 83 Tat Chee Avenue, Kowloon, Hong Kong SAR, China

[6] Key Laboratory of State Administration of Traditional Chinese Medicine of the People's Republic of China, School of Medicine, Jinan University, Guangzhou, Guangdong 510632, China

[7] These authors contributed equally.

* Corresponding author.

Prof. Yong Zhang
Provost's Chair Professor,
Department of Biomedical Engineering, College of Design and Engineering, Block E4 #04-08, 4 Engineering Drive 3, Singapore 117583, National University of Singapore
Tel: +65-65164871, Fax: +65-68723069,
Email: biezy@nus.edu.sg

Prof. Qingsong Mei
School of Medicine
Jinan University
Guangzhou, Guangdong 510632, China
E-mail: qsmei@jnu.edu.cn

Supplementary Methods

Materials and Characterization

Rare-earth oxides, Y_2O_3, Yb_2O_3, Er_2O_3, Tm_2O_3 and Nd_2O_3 were purchased from Aladdin (Shanghai, China). These rare-earth oxides were dissolved in acetic acid and kept aside for about 1 h to obtain their acetates. NaF, acetic acid and other commonly used solvents were obtained from Sinopharm Chemical Reagent Co., Ltd. (Shanghai, China). Holmium acetate hydrate (99.99%), cerium acetate hydrate (99.99%), oleic acid (OA) and octadecene (ODE) were purchased from Sigma-Aldrich. All reagents were used as received without further purification.

The morphology of nanoparticles was characterized by JEOL 2010F transmission electron microscope (Jeol Ltd., Tokyo, Japan). Upconversion emission spectra, lifetime decay curves and luminescence quantum yield of UCNPs were recorded with a fluorescence spectrometer (Edinburgh FS5 equipped with a power-adjustable 980 nm and 808 nm laser). The incident laser light was a spot shape with the diameter of 2 mm. X-ray diffraction (XRD) patterns were obtained on a Rigaku Ultimate IV diffractometer. All digital photographs were taken with a Canon EOS 70D camera. For taking photos of QR codes, the diameter of excitation light spot was amplified to about 2 cm by using of a collimator.

Synthesis of Ho/Ce co-doped UCNPs, $NaYF_4$:Yb/Ho/Ce @$NaYF_4$:Yb @$NaNdF_4$:Yb

In brief, $Ho(CH_3CO_2)_3$ (0.015mmol), $Ce(CH_3CO_2)_2$ (0.15mmol), $Yb(CH_3CO_2)_3$ (0.2mmol), $Y(CH_3CO_2)_3$ (0.635mmol), 800 mg of NaF, 20mL mixture of OA/ODE (v/v = 1:1) were added into a 100 mL three-necked round bottom flask. The mixture stirred at 110 °C in vacuum for 20min. Then it was quickly raised up to 300 °C at a rate of 10 °C/min, and kept

for 1 h in Ar_2 atmosphere to synthesis of core nanoparticles. Afterward, the first shell precursor solution was obtained by dissolving $Y(CH_3CO_2)_3$ (0.9 mmol) and $Yb(CH_3CO_2)_3$ (0.1 mmol) in 8mL mixture of OA/ODE (v/v = 1:1), and kept at 150 °C in Ar_2 atmosphere. The first shell precursor solution was dropwise added into the core reaction solution at a rate of 0.4 mL/min with the temperature of 300 °C unchanged after the core nanoparticle was successfully synthesized. Subsequently, the mixture was kept for 40 min to obtain the core@shell nanoparticles. The second shell precursor solution of $Nd(CH_3CO_2)_3$ (0.9 mmol) and $Yb(CH_3CO_2)_3$ (0.1 mmol) in 8 mL mixture of OA/ODE(v/v = 1:1) was obtained at 150 °C in Ar_2 atmosphere. Then, once the core-shell reaction was completed, it was slowly injected into the core@shell reaction solution by the similar protocol, and continued to react for 60 min. When the reaction was completed and cooled down to room temperature, equal volume of ethanol was added into the mixture. The resulted nanoparticles were precipitated and obtained through centrifugation, and washed with hexane for three times.

Synthesis of Yb/Er based UCNPs, $NaYbF_4$:Er @$NaYF_4$:Yb @$NaNdF_4$:Yb

Synthesis of $NaYbF_4$: Er^{3+} UCNPs. Typically, 0.99 mmol $Yb(CH_3CO_2)_3$, 0.01mmol $Er(CH_3CO_2)_3$, 800 mg NaF and 20 mL OA/ODE (v/v =1:1) were added into flask A, then degassed at 110 °C for 10 min and subsequently kept at 300 °C for 1 h under thorough stirring.

Synthesis of $NaYbF_4$:Er@$NaYF_4$:Yb UCNPs. A fixed amount of $Yb(CH_3CO_2)_3$, $Y(CH_3CO_2)_3$, and 8 mL OA/ODE (v/v =1:1) were added into flask B, then degassed at 110 °C for 10 min and kept stirring at 200 °C for further use. After the reaction for core nanocrystals was finished, the precursor solution in flask B was slowly injected into flask A at 300 °C, and then kept it at 300°C for 1 h.

Synthesis of $NaYbF_4$:Er@$NaYF_4$:Yb @$NaNdF_4$:Yb UCNPs. 0.1 mmol $Yb(CH_3CO_2)_3$, 0.9 mmol $Nd(CH_3CO_2)_3$, and 8 ml OA/ODE (v/v =1:1) were added into flask C, and then degassed at 110 °C for 10 min and kept stirring at 200°C for further use. After the above reaction was finished, the precursor solution in flask C was slowly injected into flask A with same protocols, and then kept it at 300 °C for 1.5 h. The final product solution was cooled to room temperature, and mixed with equal volumes of ethanol at room temperature to precipitate the nanoparticles. The final products were then isolated through centrifugation and washed with a mixture of hexane/ethanol.

Inkjet printing of QR codes

The complex QR code was prepared by inkjet printing the code of “JNU” by Yb/Er-based UCNPs as a printing ink, and code of “UCNP” by Ce/Ho-based UCNPs as another ink, at the same position of printing paper, respectively. Typically, the cyclohexane solution of Ce/Ho-based UCNPs or Yb/Er-based UCNPs was added with glycerol trioleate solution to adjust its viscosity and surface tension, obtaining well-performance printing ink. Then, the printing ink was added into two clean ink cartridges of a commercial inkjet printer. QR codes of “JNU” and “UCNP” were separately printed at the same position of A4 paper with the size of 1.5 cm × 1.5 cm. At last, luminescence patterns of the QR codes were recorded by a cannon EOS 70D camera with the integration time of 0.6 s after modulating excitation parameters.

Cautions: Only trained personnel can operate lasers. Ocular and skin exposure to laser radiation may pose the risk of eye injury and skin burns. Enclose the laser setup in a box and wear laser safety goggles at 980 nm or 808 nm when operating lasers.

Supplementary data for the UCNPs $NaYF_4$:Yb/Ho/Ce @ $NaYF_4$:Yb @ $NaNdF_4$:Yb.

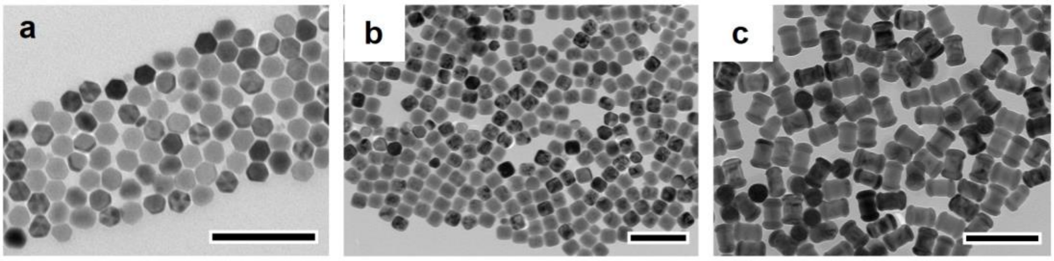

Supplementary Fig. 1. TEM images of (a) the core nanoparticles $NaYF_4$:Yb/Ho/Ce, (b) core-shell nanoparticles $NaYF_4$:Yb/Ho/Ce @ $NaYF_4$:Yb, and (c) core-shell-shell nanoparticles $NaYF_4$:Yb/Ho/Ce @ $NaYF_4$:Yb @ $NaNdF_4$:Yb. Scale bars are 50 nm.

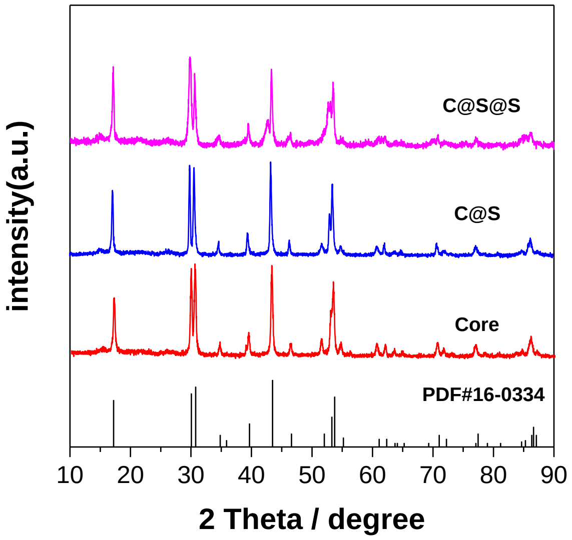

Supplementary Fig. 2. XRD patterns of the core, core-shell, and core-shell-shell nanoparticles.

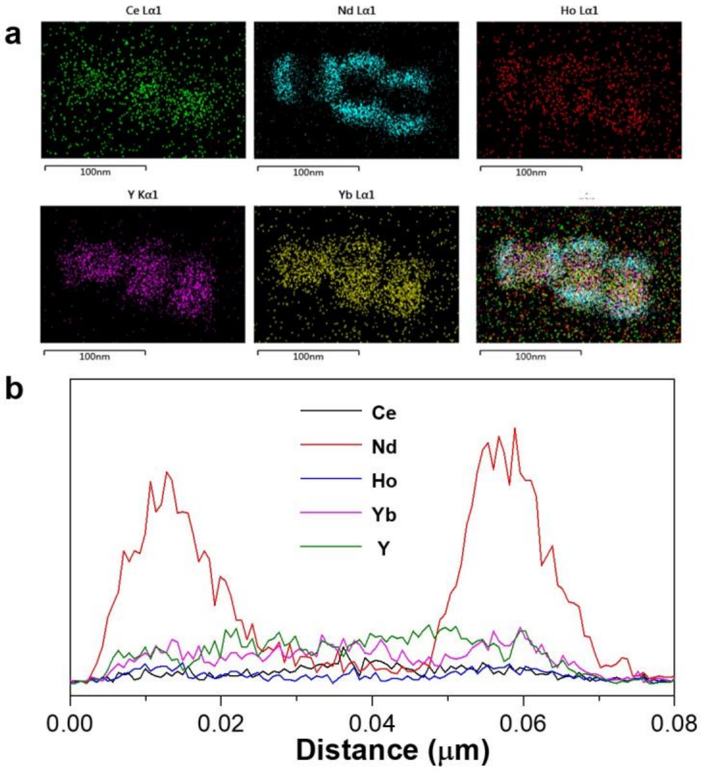

Supplementary Fig. 3. a) Elemental mapping and b) corresponding EDX line scan of Ce^{3+}, Nd^{3+}, Ho^{3+}, Y^{3+} and Yb^{3+} ions of the nanoparticles $NaYF_4$:Yb/Ho/Ce @ $NaYF_4$:Yb @ $NaNdF_4$:Yb.

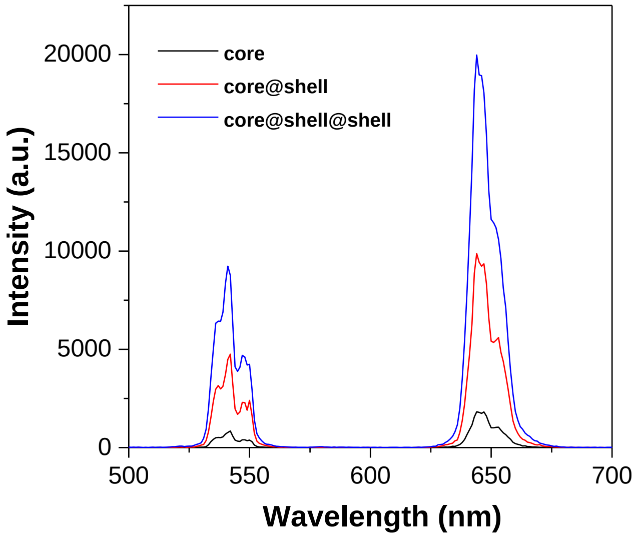

Supplementary Fig. 4. Luminescent spectra of the core nanoparticles $NaYF_4$:Yb/Ho/Ce, core-shell nanoparticles $NaYF_4$:Yb/Ho/Ce @ $NaYF_4$:Yb, and core-shell-shell nanoparticles $NaYF_4$:Yb/Ho/Ce @ $NaYF_4$:Yb @ $NaNdF_4$:Yb upon 980 nm excitation.

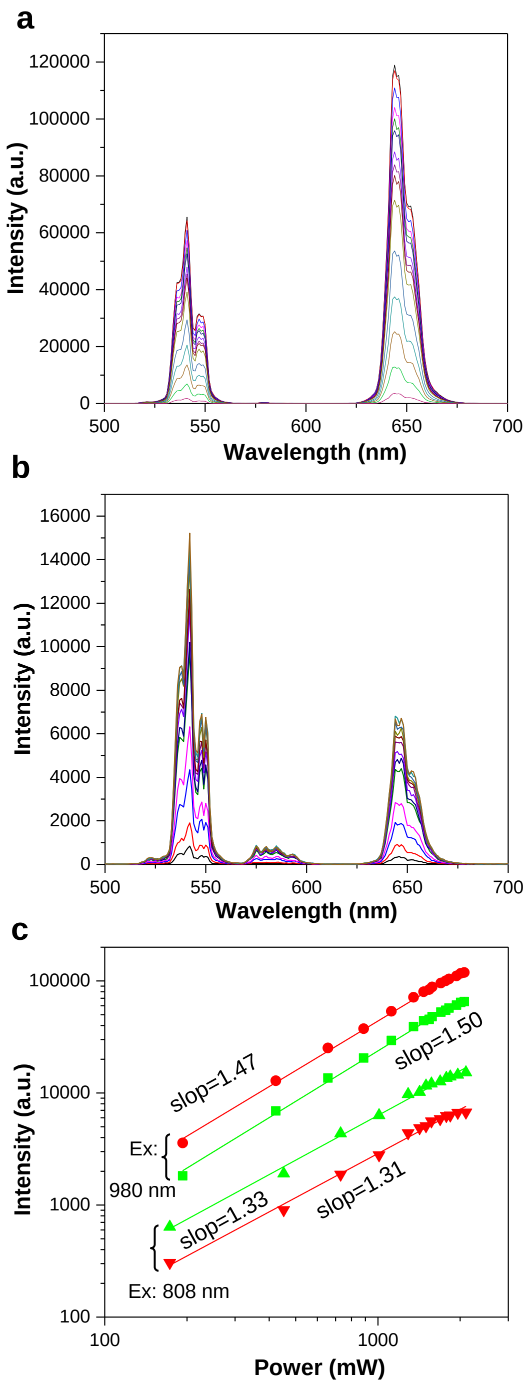

Supplementary Fig. 5. Luminescence intensity variations of the UCNP under 980 nm (a) and 808 nm (b) excitation with different power. (c) Log-log plots of the red emission (650 nm, red line) and green emission (540 nm, green line) intensities of the UCNPs toward the power of 980 nm and 808 nm lasers, respectively.

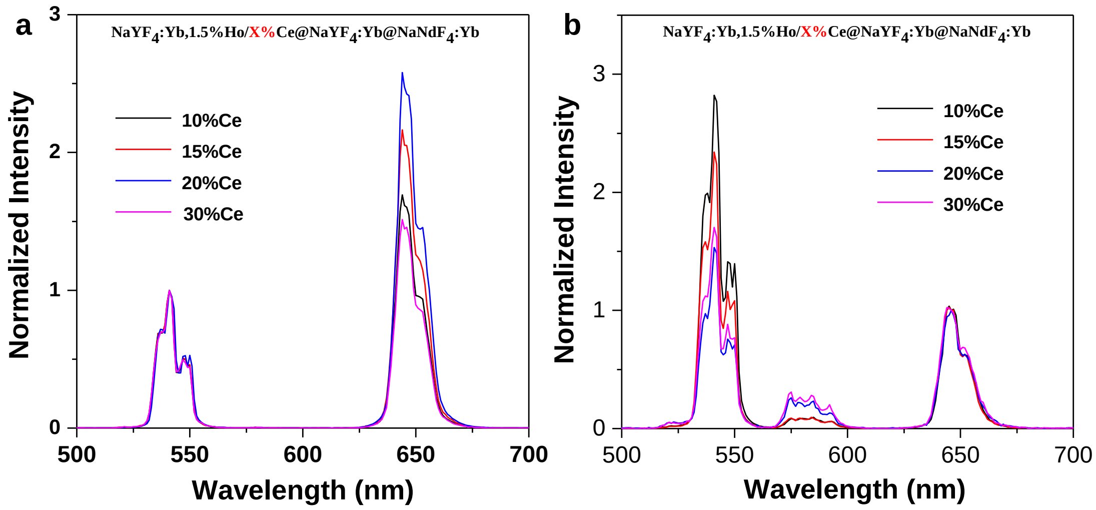

Supplementary Fig. 6. Luminescence spectra of the nanoparticles $NaYF_4$:Yb/Ho(1.5%)/Ce @ $NaYF_4$:Yb @ $NaNdF_4$:Yb with different Ce doping amounts under 980 nm (a) and 808 nm (b) excitation.

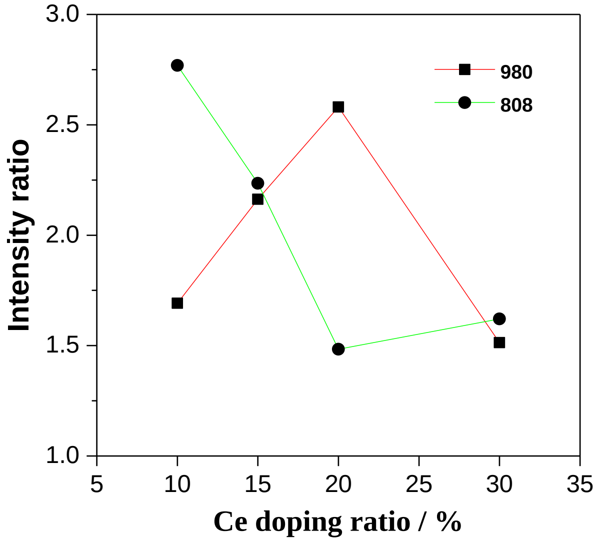

Supplementary Fig. 7. Intensity ratios of red emission to green emission of the nanoparticles $NaYF_4$:Yb/Ho(1.5%)/Ce(x%) @ $NaYF_4$:Yb @ $NaNdF_4$:Yb at 980 nm excitation, and ratios of green to red at 808 nm excitation.

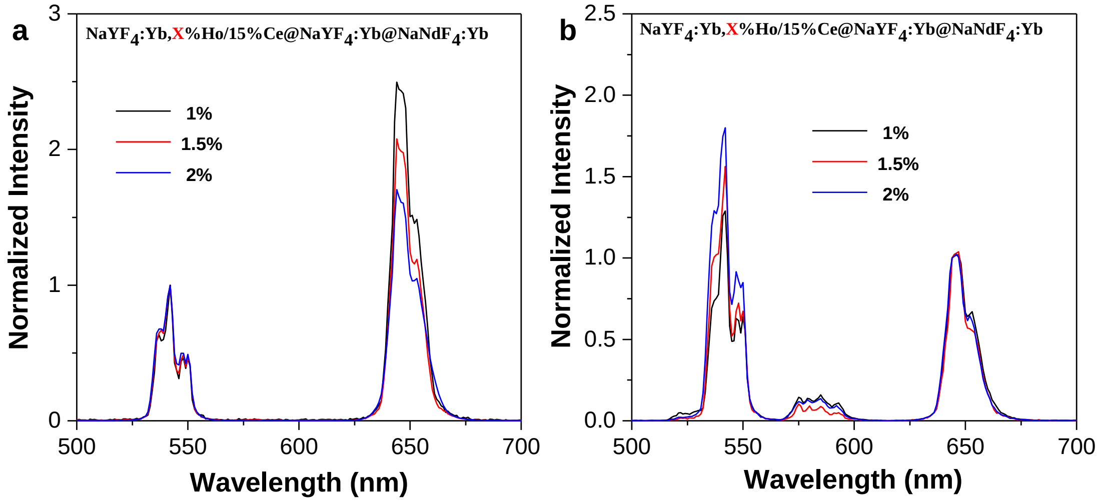

Supplementary Fig. 8. Luminescence spectra of the nanoparticles $NaYF_4$:Yb/Ho/Ce(15%) @ $NaYF_4$:Yb @ $NaNdF_4$:Yb with different Ho doping amounts under 980 nm (a) and 808 nm (b) excitation.

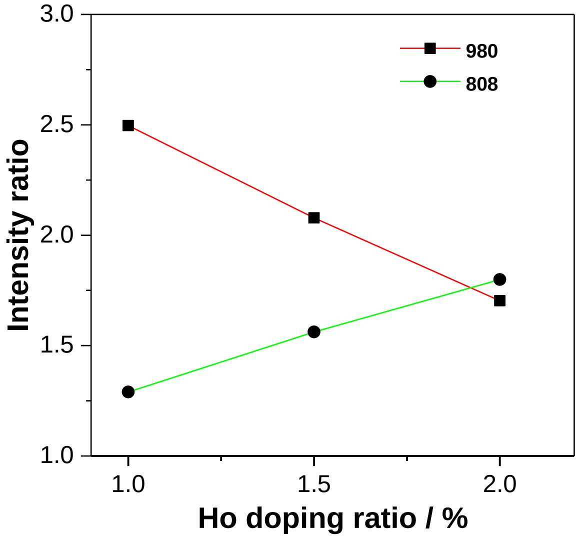

Supplementary Fig. 9. Intensity ratios of red emission to green emission of the nanoparticles $NaYF_4$:Yb/Ho(x%)/Ce(15%) @ $NaYF_4$:Yb @ $NaNdF_4$:Yb at 980 nm excitation, and ratios of green to red at 808 nm excitation.

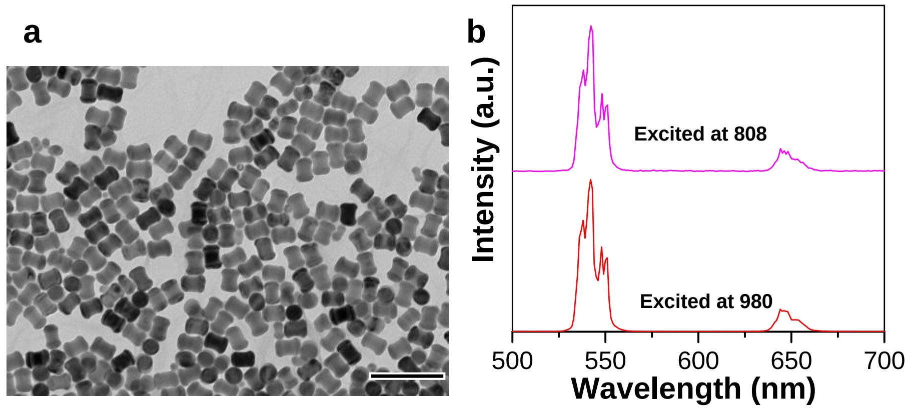

Supplementary Fig. 10. (a) TEM image and (b) luminescence spectra of the nanoparticles $NaYF_4$:Yb/Ho @ $NaYF_4$:Yb @ $NaNdF_4$:Yb under 980 nm and 808 nm excitation.

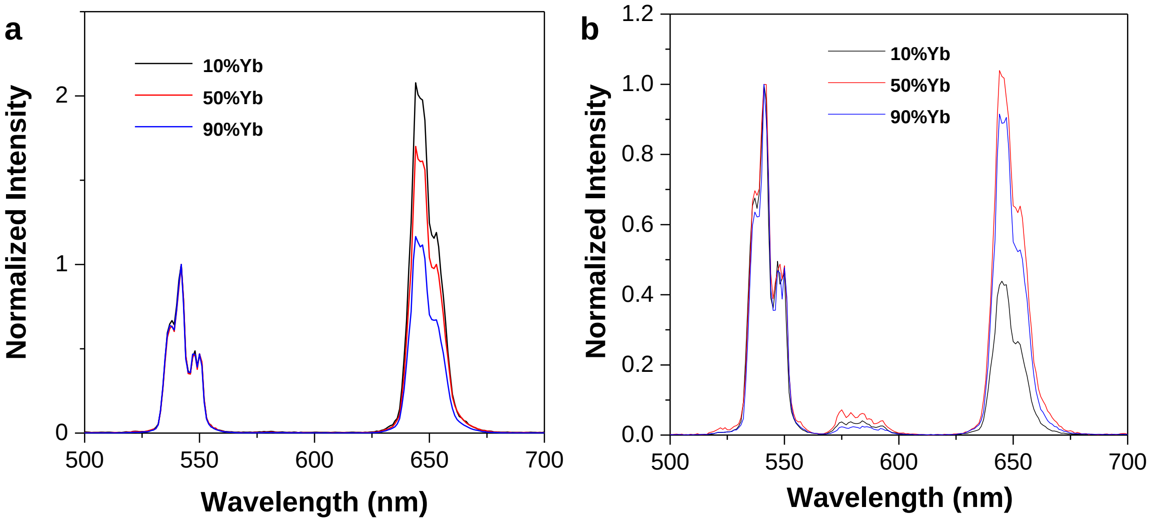

Supplementary Fig. 11. Luminescence spectra of the nanoparticles $NaYF_4$:Yb/Ho(1.5%)/Ce(15%) @ $NaYF_4$:Yb(x%) @ $NaNdF_4$:Yb with different Yb doping amounts in migrating shell under 980 nm (a) and 808 nm (b) excitation.

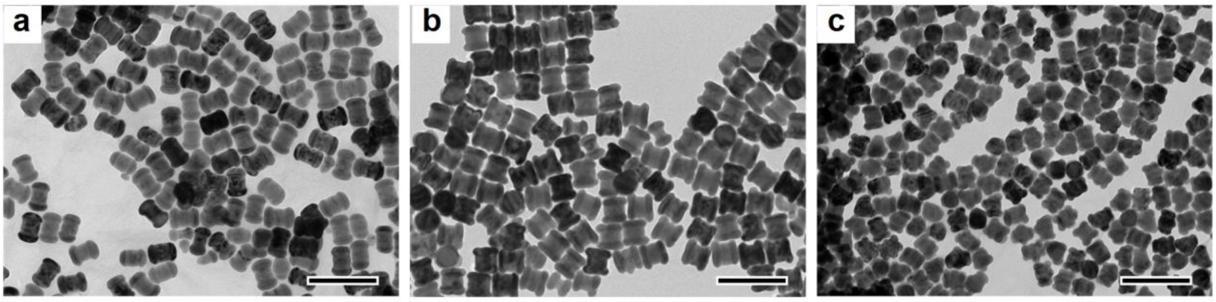

Supplementary Fig. 12. TEM images of the nanoparticles $NaYF_4$:Yb/Ho(1.5%)/Ce(15%) @ $NaYF_4$:Yb(x%) @ $NaNdF_4$:Yb with different Yb doping amounts in migrating shell, (a) 10%, (b) 50%, (c) 90%. Scale bars are 200 nm.

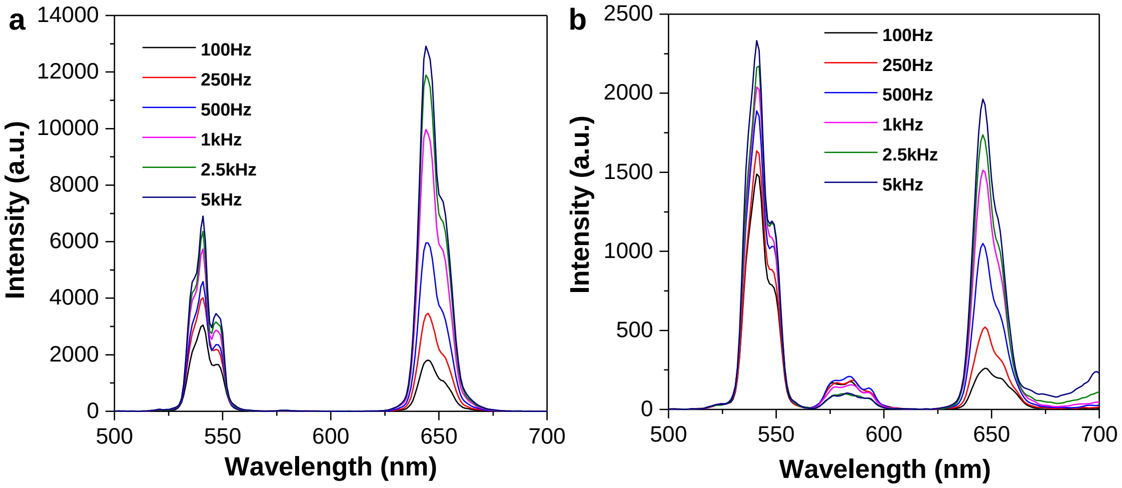

Supplementary Fig. 13. Luminescence spectra of the nanoparticles $NaYF_4$:Yb/Ho(1.5%)/Ce(15%) @ $NaYF_4$:Yb @ $NaNdF_4$:Yb excited with 980 nm (a) and 808 nm (b) laser with different frequencies.

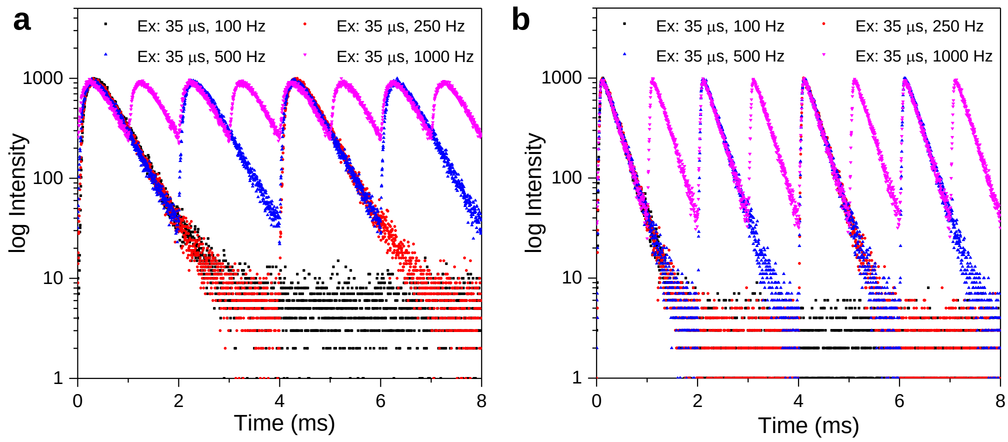

Supplementary Fig. 14. Time-resolved luminescence investigations of red emission at 650 nm (a), and green emission at 540 nm (b) of $NaYF_4$:Yb/Ho/Ce @ $NaYF_4$:Yb @ $NaNdF_4$:Yb upon changing 980 nm excitation frequency from 100 Hz to 1000 Hz.

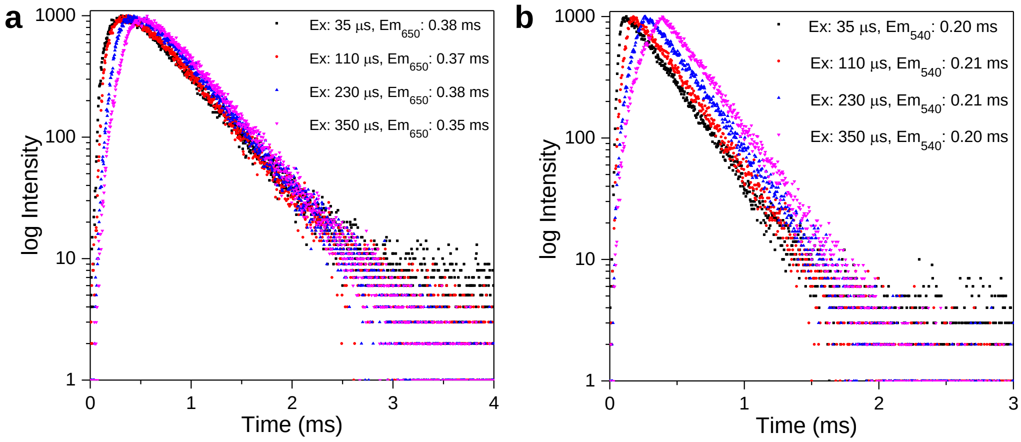

Supplementary Fig. 15. Time-resolved luminescence investigations of red emission at 650 nm (a), and green emission at 540 nm (b) of $NaYF_4$:Yb/Ho/Ce @ $NaYF_4$:Yb @ $NaNdF_4$:Yb upon changing 980 nm pulse width from 35 μs to 350 μs with the frequency of 100 Hz.

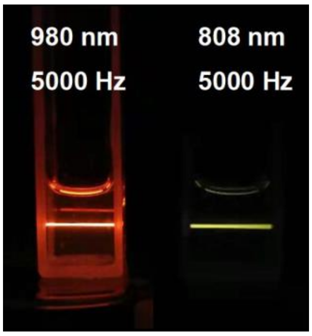

Supplementary Fig. 16. Luminescent images of the nanoparticles $NaYF_4$:Yb/Ho(1.5%)/Ce(15%) @ $NaYF_4$:Yb @ $NaNdF_4$ upon irradiation at a 980 nm or 808 nm pulse laser with frequency of 5000 Hz.

Supplementary data for the UCNPs $NaYbF_4$:Er @ $NaYF_4$:Yb @ $NaNdF_4$:Yb.

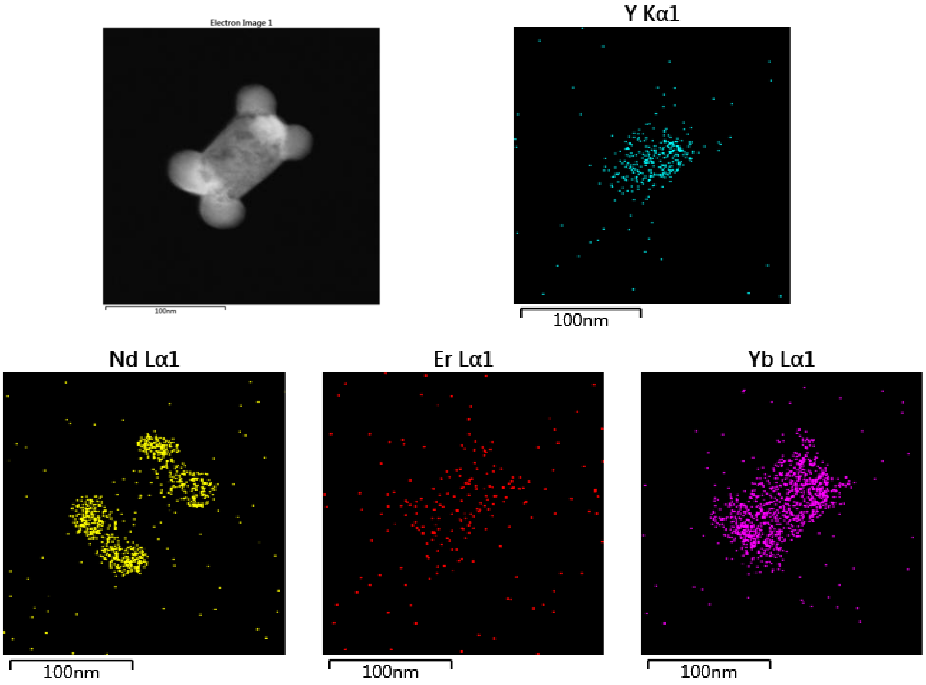

Supplementary Fig. 17. Elemental mapping of Y^{3+}, Nd^{3+}, Er^{3+} and Yb^{3+} ions of the nanoparticles $NaYbF_4$:Er @ $NaYF_4$:Yb (10%) @ $NaNdF_4$:Yb.

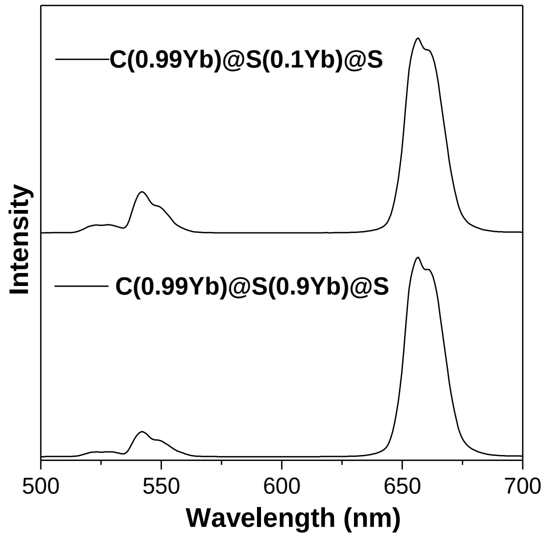

Supplementary Fig. 18. Luminescent spectra of the nanoparticles $NaYbF_4$:Er @ $NaYF_4$:Yb (x%) @ $NaNdF_4$:Yb under 980 nm excitation when Yb doping ratio changed from 10% to 90% in the migrating shell.

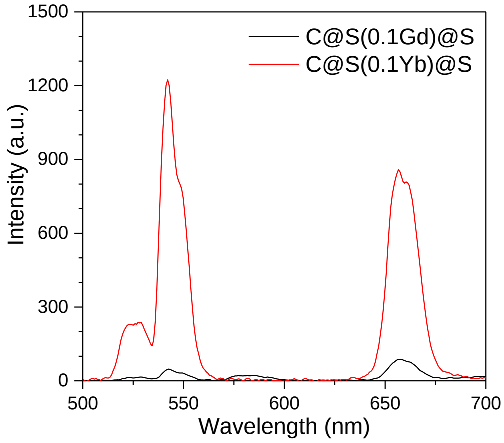

Supplementary Fig. 19. Luminescence comparison between $NaYbF_4$:Er @ $NaYF_4$:Gd(10%) @ $NaNdF_4$:Yb (black line) and $NaYbF_4$:Er @ $NaYF_4$:Yb(10%) @ $NaNdF_4$:Yb (red line) under excitation with 808 nm.

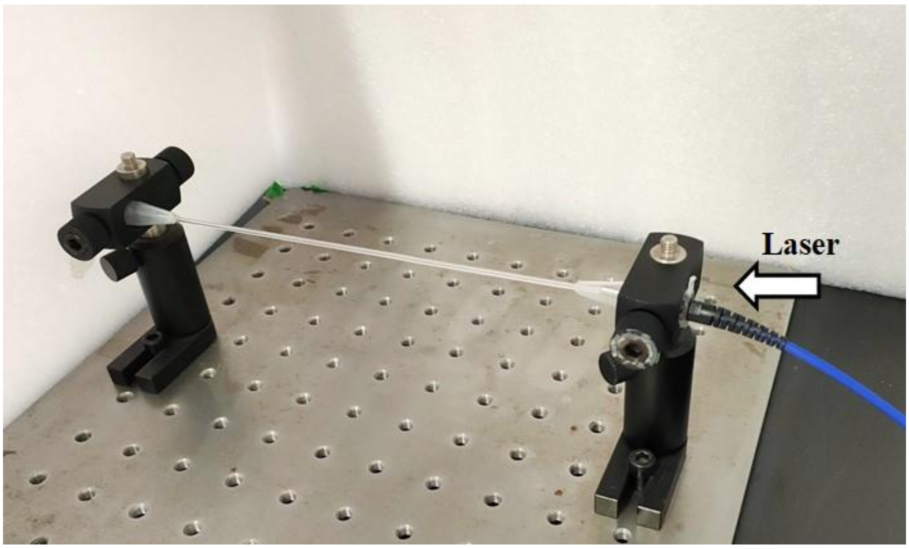

Supplementary Fig. 20. The setup photo of quartz capillary to exhibit luminescence variations of different UCNPs under 980 nm or 808 nm laser excitations.

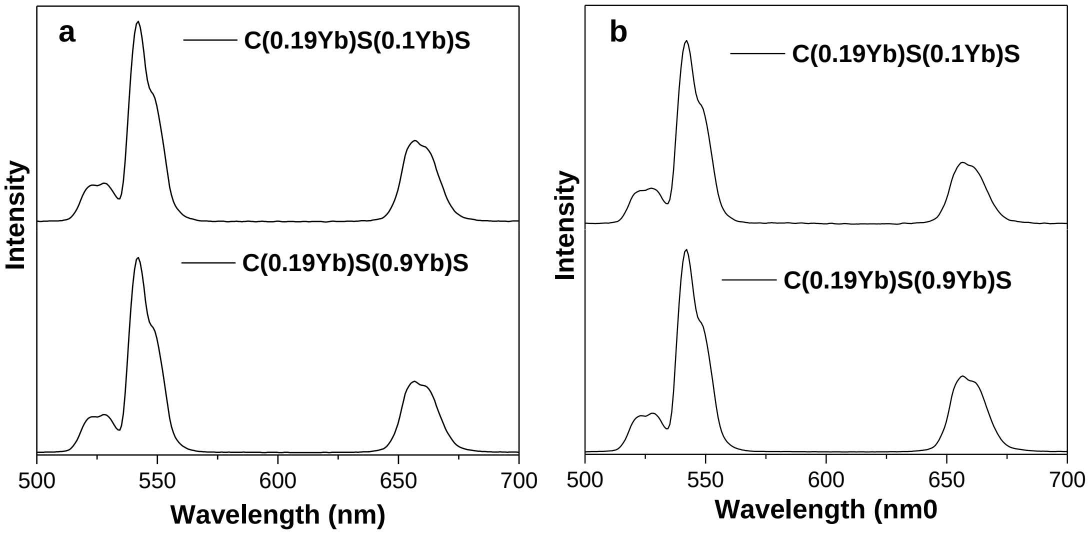

Supplementary Fig. 21. Luminescent spectra of the nanoparticles $NaYF_4$:Yb(19%)/Er(1%) @ $NaYF_4$:Yb (x%) @ $NaNdF_4$:Yb under 980 nm (a) and 808 nm (b) excitation.

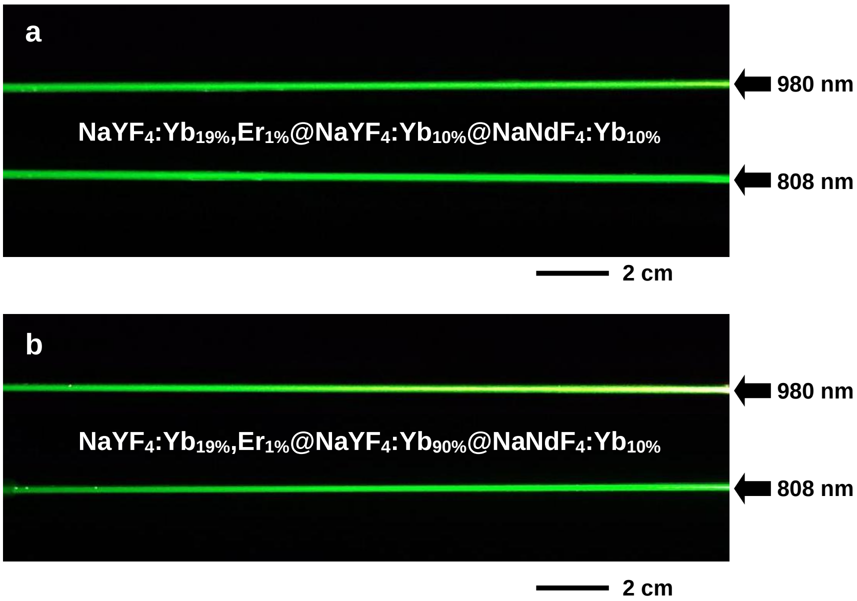

Supplementary Fig. 22. (a) Luminescent images of the nanoparticles $NaYF_4$:Yb(19%)/Er(1%) @ $NaYF_4$:Yb (10%) @ $NaNdF_4$:Yb in capillary under 980 nm and 808 nm excitation. b) Luminescent images of the nanoparticles $NaYF_4$:Yb(19%)/Er(1%) @ $NaYF_4$:Yb (90%) @ $NaNdF_4$:Yb in capillary under 980 nm and 808 nm excitation. Scale bar is 2 cm.

Supplementary Table 1. Luminescent color comparisons of the Yb-Er based UCNPs at different excitation conditions.

	980 nm		808 nm	
	1.7 W	4.8 W	1.7 W	4.8 W
$NaYbF_4$:Er @ $NaYF_4$:Yb(10%) @ $NaNdF_4$:Yb	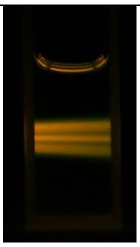	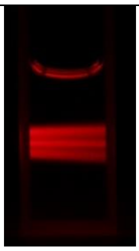	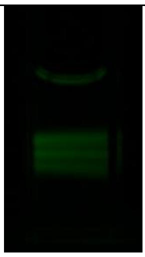	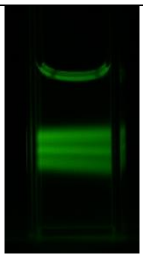
$NaYbF_4$:Er @ $NaYF_4$:Yb(90%) @ $NaNdF_4$:Yb	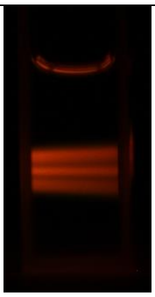	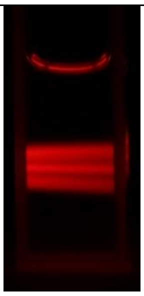	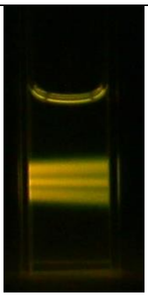	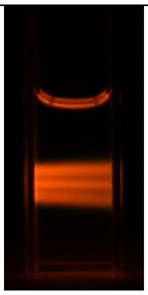

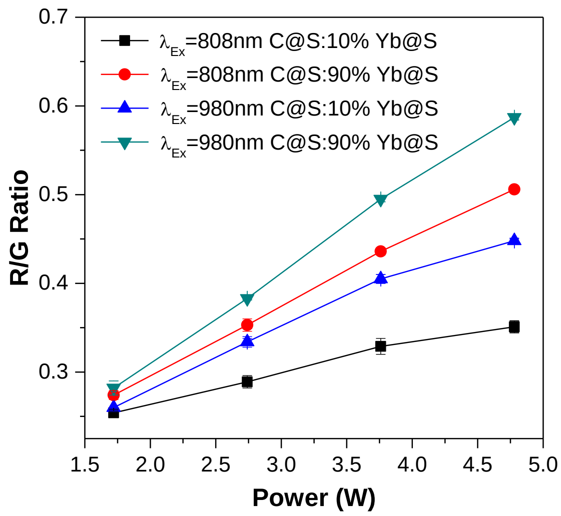

Supplementary Fig. 23. Intensity ratios of red emission to green emission of the nanoparticles $NaYF_4$:Yb(19%)/Er(1%) @ $NaYF_4$:Yb (x%) @ $NaNdF_4$:Yb with different 980 nm and 808 nm excitation power. Data are presented as mean values ±SD (n=3).

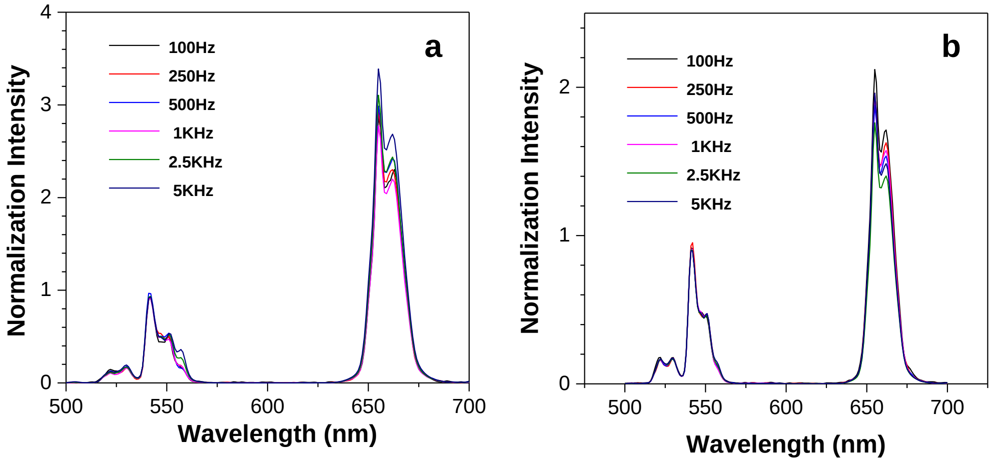

Supplementary Fig. 24. Luminescence spectra of the nanoparticles $NaYbF_4$:Er @ $NaYF_4$:Yb (90%) @ $NaNdF_4$:Yb excited with 980 nm (a) and 808 nm (b) laser with different frequencies.

Supplementary Table 2. Table list of the detail meaning of each excitation parameters.

**Value**	1	0
**x (λ)**	980 nm	808 nm
**y (f)**	High frequency (>1000 Hz)<br>(2500 Hz used in Figure 4)	Low frequency (<1000 Hz)<br>(100 Hz used in Figure 4)
**z (P)**	High power (> 0.5 W)<br>(3 W/cm^2 used in Figure 4)	Low power (< 0.5 W)<br>(0.5 W/cm^2 used in Figure 4)

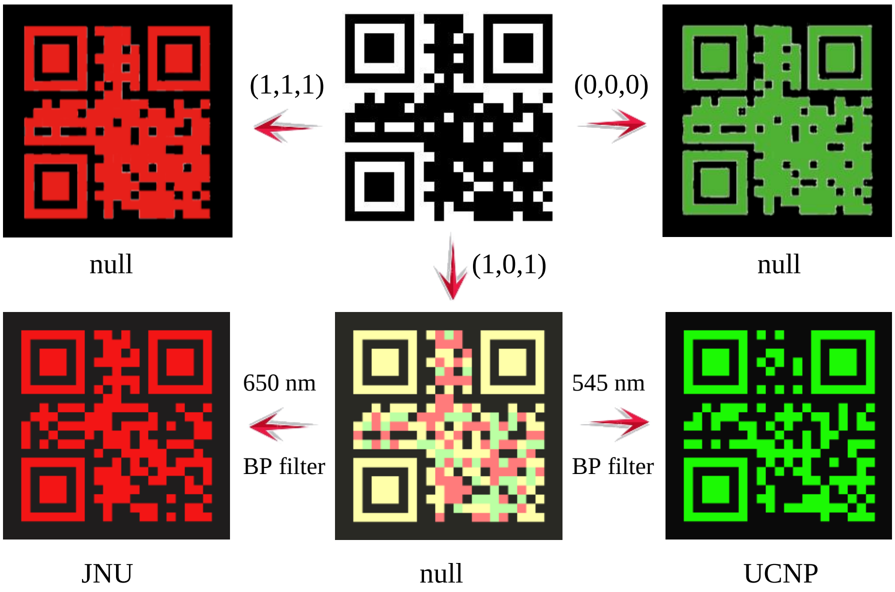

Supplementary Fig. 25. Proof-of-concept demonstration of three-dimensional decryption of the luminescent QR code at specific excitation parameters. These QR codes were computer-generated.

Supplementary Table 3. Absolute upconversion quantum yields of the Ce/Ho-based and Yb/Er-based UCNPs.

Sample	Quantum Yield (%)	Excitation Wavelength (nm)	Excitation Power (W)
Ce/Ho-based UCNPs	0.93<br>0.68	980<br>808	2.1<br>2.1
Yb/Er-based UCNPs	0.92<br>0.53	980<br>808	2.1<br>2.1
$NaYF_4$:Yb/Er (18/2%)	0.37	980	2.1
$NaYF_4$:Yb/Er/Nd (18/2/2%) @ $NaYF_4$:Nd	0.35	808	2.1

We have measured quantum yields of the Ce/Ho-based and Yb/Er-based UCNPs at 980 nm and 808 nm laser excitation. It is found from Table R-1 that these measured quantum yield values are comparable to the typically-used UCNPs, $NaYF_4$:Yb/Er (18/2%) and $NaYF_4$:Yb/Er/Nd (18/2/2%) @ $NaYF_4$:Nd (20%), respectively exited with 980 nm and 808 nm irradiations.

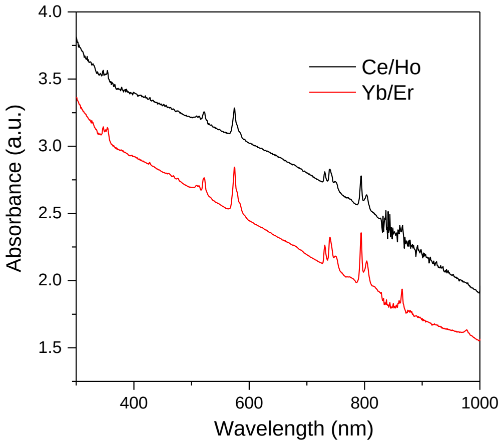

Supplementary Fig. 26. The absorption spectra of Ce/Ho-based UCNPs and Yb/Er-based UCNPs with the concentrations of 10 mg/mL.
